# Association between treatment with sacubitril/valsartan and the risk of Alzheimer’s disease: a clinical update

**DOI:** 10.1186/s13195-024-01547-z

**Published:** 2024-08-01

**Authors:** Antoine Garnier-Crussard

**Affiliations:** 1 Clinical and Research Memory Centre of Lyon, Lyon Institute For Aging, Hospices Civils de Lyon, Hôpital des Charpennes, 27 rue Gabriel Péri, Villeurbanne, 69100 France; 2 Normandie Univ, UNICAEN, INSERM, U1237, PhIND “Physiopathology and Imaging of Neurological Disorders”, Neuropresage Team, Cyceron, Caen, 14000 France

**Keywords:** Sacubitril/valsartan, Entresto, Neprilysin, LCZ696, Amyloid, Alzheimer

## Abstract

Since 2014, sacubitril/valsartan (Entresto^®^) is widely prescribed for heart failure. Despite neprilysin inhibition’s benefits in heart failure, concerns about potential amyloid-beta (Aβ) accumulation and Alzheimer’s disease (AD) risk have persisted. This narrative review, a decade post-approval, evaluates the risk of amyloid pathology and neurocognitive disorders in long-term sacubitril/valsartan use. Clinical trials, real-world studies, and pharmacovigilance data do not indicate an increased risk of cognitive decline. In patients treated with sacubitril/valsartan blood-based amyloid biomarkers show perturbations, while neuroimaging biomarkers reveal no significant increase in amyloid load. Despite a theoretical risk of amyloid accumulation and AD under treatment with sacubitril/valsartan, current clinical data appears reassuring, and there is no signal indicating an increased risk of cognitive decline, but a perturbation of amyloid blood-based biomarkers, which implies great caution when interpreting biomarkers in this context.

## Background

Since the findings of PARADIGM-HF study in 2014, sacubitril/valsartan (LCZ696, Entresto^®^) is now recommended and commonly prescribed for heart failure (HF), including in older adults [1, 2]. Worldwide, it is estimated that more than 2.8 million patients are treated with sacubitril/valsartan [3]. Specifically in 2021 in the United States of America, 394,848 Medicare beneficiaries were treated with sacubitril/valsartan [4].

LCZ696 (sacubitril/valsartan) contains a combination of sacubitril and valsartan. Valsartan is an angiotensin II receptor blocker, used alone or associated with other medicines to treat hypertension or heart failure notably. Sacubitril is a neprilysin inhibitor, a metalloprotease inactivating, the natriuretic peptide. However, neprilysin also targets other proteins, including the amyloid-beta (Aβ) peptide implicated in Alzheimer’s disease (AD). The involvement of neprilysin in AD pathophysiology has been documented since the 2000s [5, 6], and its role in amyloid clearance is established [7]. Thus, while neprilysin inhibition has a beneficial effect on HF, it could theoretically be associated with a risk of limiting Aβ peptide degradation, and thereby increasing the risk of AD. As early as 2014, concerns about this potential risk were raised [8–12]. This narrative review aims to assess the risk of amyloid pathology accumulation and the risk of neurocognitive disorders in patients treated with long-term sacubitril/valsartan, a decade after the publication of the PARADIGM-HF study results.

## Main text

### Association between sacubitril/valsartan and cognitive decline

In the PARADIGM-HF study, a secondary analysis investigating differences in adverse events related to dementia showed no difference between the sacubitril/valsartan group and the enalapril group after 27 months [13]. The PARAGON-HF study, which assessed the efficacy of sacubitril/valsartan in HF with preserved ejection fraction, pre-specified an analysis of cognition in treated patients. Cognition was assessed through successive measurements of the Mini Mental State Examination, which is not a sensitive tool to detect early and mild cognitive changes [14]. The study found no difference in cognitive decline between the two groups at 96 weeks [15]. More recently, in line with FDA requirements, the PERSPECTIVE study, a multicenter randomized trial assessing the long-term neurocognitive effects and safety of sacubitril/valsartan, was conducted. This trial included 592 patients (mean age 72.4 years) randomized between sacubitril/valsartan and valsartan. This study did not show an increased risk of cognitive disorders (cognition assessed by the CogState global cognition composite score) at 36 months in patients treated by sacubitril/valsartan (ESC 2022 Congress [16]).

Beyond these trials, real-world database studies have investigated the relationship between treatment with sacubitril/valsartan and the risk of cognitive decline or neurocognitive disorders. A study using electronic health record databases compared the risk of developing a neurocognitive disorder between a group of patients treated with sacubitril/valsartan and a matched group. The groups (*N* = 19,553 per group, mean age 63 years) were balanced, and the authors showed that the incidence of neurocognitive disorders was lower in the sacubitril/valsartan group compared to the control group [17]. By extension, the same team subsequently used the same methodology to investigate the risk of cognitive decline after 3 years of exposure (*N* = 11,313 per group) and seemed to confirm the lower risk of neurocognitive disorders in patients on sacubitril/valsartan [18]. Similarly, a study using the Korean National Health Insurance Service database, which included 6,789 patients on sacubitril/valsartan and 13,578 controls, did not find an increased risk of neurocognitive disorder in the sacubitril/valsartan group after a mean follow-up of 2.5 years [19].

Beyond clinical studies, pharmacovigilance data provide valuable insights into the potential risk induced by drug exposure [20, 21], and a recent query on the FDA Adverse Event Reporting System pharmacovigilance data identified 80,316 notifications of sacubitril/valsartan in HF patients. The authors did not find a significantly higher reporting of neurocognitive disorders [20].

### Association between sacubitril/valsartan and Alzheimer’s biomarkers

A randomized controlled study involving 42 healthy participants (mean age 38 years), did not find a difference in amyloid peptide Aβ1–40 and Aβ1–42 levels (isolated increase in Aβ1–38) in cerebrospinal fluid but observed an increase in amyloid peptide Aβ1–40 in the blood after 14 days of exposure to sacubitril/valsartan [22]. A recent study measured the evolution of blood biomarkers for AD with blood samples from a previous randomized controlled trial [23]. Ninety-two patients were included (mean age 61 years). The authors observed a significant elevation in plasma Aβ40 and Aβ42 levels at 26 and 52 weeks of treatment, with a decrease in the Aβ42/Aβ40 ratio by approximately 30% in the sacubitril/valsartan group. Other plasma biomarkers, including phosphorylated tau proteins (p-tau217 and p-tau181, reflecting both tau and amyloid pathology [24]), did not show a significant change under treatment. The authors hypothesize that the differences in Aβ40 and Aβ42 with sacubitril/valsartan is explained by the reduced peripheral neprilysin activity [23]. Sacubitril metabolite crosses the blood-brain barrier in healthy subjects and may inhibit neprilysin, but to a small extent [22]. The peripheral and central effects of neprilysin on amyloid metabolism need to be further explored, including in older adults with possible blood-brain barrier dysfunction. In any case, these studies call for the utmost caution in interpreting blood amyloid biomarkers in patients treated with sacubitril/valsartan. As direct-to-consumer AD blood test (plasma Aβ42/Aβ40) has arrived [25], there is an increased risk of misleading results in patients treated with sacubitril/valsartan, that could have very negative consequences for patients and highlight the need of expert guidance for the interpretation of blood-based biomarkers in this context.

Finally, the PERSPECTIVE study assessed the evolution of cerebral amyloid load measured by positron emission tomography at 3-year in 491 patients. Authors showed no significant difference in the variation of amyloid tracer uptake in patients treated with sacubitril/valsartan [16].

## Conclusion

Despite a theoretical risk of amyloid accumulation and AD under treatment with sacubitril/valsartan, current clinical data, derived from interventional trials, observational studies using real-world data, or pharmacovigilance registries are reassuring, and there is no signal indicating an increased risk of cognitive decline, but a perturbation of amyloid blood-based biomarkers.

**Fig. 1:**
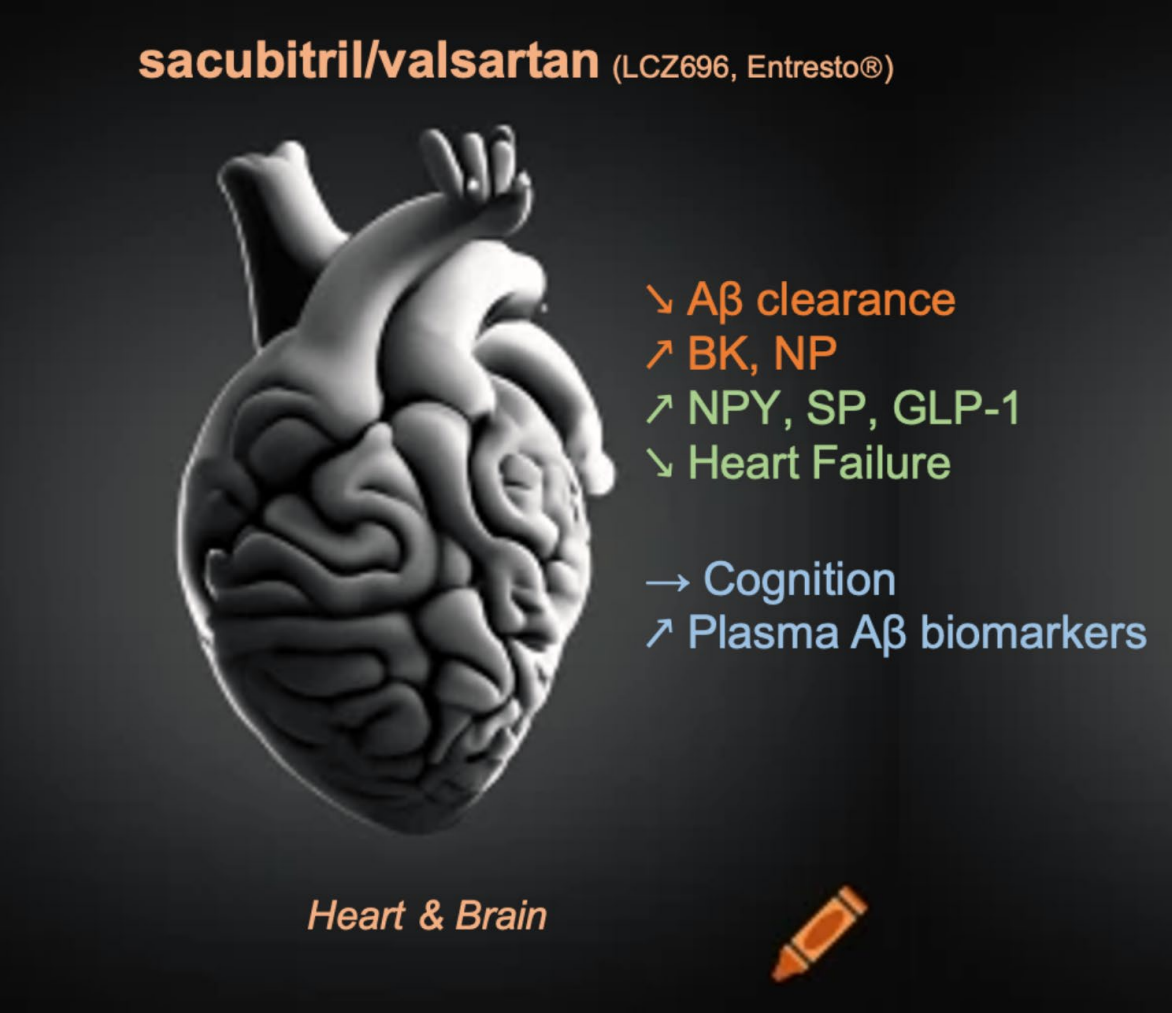
Summary of the effect of sacubitril/valsartan and AD risk. Neprilysin inhibition (sacubitril) may lead to both a potential and theoretical protective pathway (e.g., enhancing neuroprotective neuropeptide Y (NPY), substance P (SP), and glucagon-like peptide 1 (GLP-1), and improving heart function - green text in the figure) and a pathway potentially/theoretically increasing the risk of AD (e.g., reducing Aβ clearance, elevating bradykinin (BK), and natriuretic peptides (NP)… - orange text in the figure) [7]. To date, there is no clinical evidence in humans indicating an elevated risk of cognitive decline or Alzheimer’s disease during treatment, but there are alterations in amyloid blood-based biomarkers (blue text in the figure). Image Heart & Brain generated/drawn by AI (https://www.craiyon.com), and edited by A.G-C.

The absence of an elevated risk of neurocognitive disorders with sacubitril/valsartan can be explained by several hypotheses. Firstly, sacubitril metabolite crosses the blood-brain barrier but to a small extent [22], and no significant cerebral amyloid pathology was observed in treated patients [16, 22]. Secondly, the relationships between neprilysin inhibition and Alzheimer’s pathology are complex and cannot be limited to the peptidase effect on the amyloid protein. Beyond the theoretical inhibition of Aβ peptide clearance, other potentially AD-risk pathways exist, such as increased bradykinin and natriuretic peptides [7], and also potentially protective pathways, such as increased neuroprotective peptides (neuropeptide Y, substance P, glucagon-like peptide 1 GLP-1) [7]. Thirdly, the cardiovascular effect of sacubitril/valsartan could contribute to improving vascular health and reducing the risk of neurocognitive disorders [26] (Figure 1).

While the clinical data to date are reassuring and even potentially suggest a protective effect of sacubitril/valsartan, caution is necessary, especially regarding the potential risk highlighted by animal studies in chemical induced rat models [27, 28]. In these studies, sacubitril/valsartan treatment caused deleterious effect on cognition in rats while valsartan alone did not. Moreover, potential long-term effects of sacubitril/valsartan need to be monitored. Indeed, amyloid deposition can precede the clinical symptoms of AD by more than 10 years [29], necessitating long-term follow-up with neuropsychological tools sensitive to cognitive changes in preclinical or prodromal AD, and possible individual assessment of AD risk including genetic risk profiles in the future [30]. Finally, while plasma biomarkers are expected to be beneficial in detecting AD in the future, special attention will be required for plasma amyloid biomarkers, which may be inconclusive in patients treated by sacubitril/valsartan treatment [23].

## Data Availability

No datasets were generated or analysed during the current study.

## Abbreviations

AD: Alzheimer’s disease
ESC: European Society of Cardiology
FDA: Food and Drug Administration
HF: Heart failure
MMSE: Mini Mental State Examination
p-tau: phosphorylated tau protein
